# Controversies in NEN: An ENETS position statement on the endoscopic management of localised gastric, duodenal and rectal neuroendocrine neoplasms

**DOI:** 10.1111/jne.70060

**Published:** 2025-06-16

**Authors:** Francesco Panzuto, Dermot O'Toole, Günter Klöppel, Ulrich Peter Knigge, Günter Josef Krejs, Marina Tsoli, Marco Volante, Tu Vinh Luong

**Affiliations:** ^1^ Department of Medical‐Surgical Sciences and Translational Medicine Sapienza University of Rome Rome Italy; ^2^ Digestive Disease Unit, ENETS Center of Excellence Sant'Andrea University Hospital Rome Italy; ^3^ Department of Gastroenterology St James Hospital, ENETS Center of Excellence, St Vincent's University Hospital and Trinity College Dublin Ireland; ^4^ Department of Pathology School of Medicine and Health, Technical University Munich Munich Germany; ^5^ Neuroendocrine Tumor Unit, ENETS Center of Excellence, Department of Transplantation and Digestive Diseases and Department of Nephrology and Endocrinology Rigshospitalet, University of Copenhagen Copenhagen Denmark; ^6^ Department of Internal Medicine Medical University of Graz Graz Austria; ^7^ Neuroendocrine Tumor Unit, ENETS Centre of Excellence, 1st Department of Propaedeutic Internal Medicine Laiko Hospital, National and Kapodistrian University of Athens Athens Greece; ^8^ Department of Oncology University of Turin, at San Luigi Hospital, Orbassano Turin Italy; ^9^ Neuroendocrine Tumor Unit, ENETS Center of Excellence, Department of Cellular Pathology Royal Free London NHS Foundation Trust London UK

**Keywords:** endoscopic resection, endoscopic ultrasonography, guidelines, management, neuroendocrine tumours

## Abstract

Gastric, duodenal and rectal neuroendocrine tumours (NETs) are increasingly detected due to advances in endoscopic imaging. While international guidelines provide criteria for endoscopic management, several aspects remain controversial due to limited high‐quality evidence. This position paper, developed by an expert panel, aims to clarify these unresolved issues and provide consensus‐based recommendations. The primary objective of this position paper is to critically analyse and address key controversies in the endoscopic management of gastro‐duodenal‐rectal NETs. These include the optimal selection of endoscopic resection techniques, the significance of R1 resections, pathological assessment and surveillance strategies. Special attention is given to site‐specific challenges, including the role of Ki‐67 in type 1 gastric NETs, the management of multiple gastric lesions, the feasibility of endoscopic resection for type 3 gastric NETs and the limitations of advanced endoscopic techniques in the duodenum. This position paper was developed using an Expert Panel Consensus methodology. Topics were identified during the 2024 ENETS Advisory Board meeting and addressed through a structured literature review. Evidence was critically appraised, and expert discussions were conducted to identify key points. By reviewing controversial aspects of endoscopic management, this position paper will provide practical guidance to optimise decision‐making and improve outcomes for patients with gastro‐duodenal‐rectal NETs. Multidisciplinary evaluation remains crucial to tailoring treatment strategies based on tumour characteristics, patient factors and procedural risks.

## 1 INTRODUCTION

Gastric, duodenal and rectal neuroendocrine neoplasms (NENs) have markedly increased in incidence and prevalence over the last decades.^1^ Like all digestive NENs, they are classified into well‐differentiated (neuroendocrine tumours – NETs) and poorly differentiated (neuroendocrine carcinomas – NECs). The well‐differentiated category is further divided based on their Ki‐67 labelling index or mitotic count into G1 (Ki‐67 <3% or mitoses <2/HPF), G2 (Ki‐67 3%–20% or mitoses 2‐20/HPF) and G3 (Ki‐67 >20% or mitoses >20/HPF).^2^ Gastric NETs are also classified clinically, depending on concurrent gastric pathology and elevated gastrin levels.^3^ Thus, we identify type 1 gastric NETs, associated with chronic atrophic gastritis (CAG) of the gastric body and a vitamin B12 deficiency; type 2, associated with Zollinger‐Ellison syndrome in the context of Multiple Endocrine Neoplasia type 1 (MEN1); and sporadic type 3, when there is no associated pathology and normal blood gastrin. According to the ENETS recommendations,^4^, ^5^ most gastric, duodenal and rectal NETs (gNETs, dNETs, rNETs) are managed through an endoscopic therapeutic approach, provided certain conditions are met (Table 1). However, even within the context of these recommendations, some aspects remain unclear and disputed, since the supporting literature is either sparse or of low scientific quality. For this reason, this position paper aims to thoroughly address and discuss those aspects considered controversial in the endoscopic management of gastro‐duodenal‐rectal NETs.

**TABLE 1:** Indication for endoscopic resection in gNETs, dNETs, and rNETs.

	<1 cm	1–2 cm	>2 cm
Gastric NETs	Type 1: Not always necessary Type 3: Advisable, if grade 1 NET	Type 1: Recommended through EMR/ESD Type 3: Usually not recommended (possible in very selected low‐grade cases)	Type 1: Usually not recommended (possible in very selected low‐grade cases) Type 3: Not recommended
Duodenal NETs	Recommended, if non‐functioning non‐ampullary NET through EMR (ESD to be used with extreme caution)	Usually not recommended (possible in very selected low‐grade cases: see chapter 3.5.2 and 3.5.3)	Not recommended
Rectal NETs	Recommended through EMR/ESD	Usually not recommended (possible in selected low‐grade cases through ESD)	Not recommended

## 2 MATERIALS AND METHODS

This position paper was developed through expert panel consensus, leveraging the collective expertise of leading specialists to address controversial aspects of gastro‐duodenal‐rectal NETs. Key issues were identified during the ENETS Advisory Board meeting in November 2024.

After topic selection, a non‐systematic review was conducted via PubMed using the following search strategy ((‘Gastrointestinal Neuroendocrine Tumors’ OR ‘Rectal Neuroendocrine Tumors’ OR ‘Gastric Neuroendocrine Tumors’ OR ‘Duodenal Neuroendocrine Tumors’ OR ‘gastroenteropancreatic (GEP)‐NEN’). AND (‘Endoscopy’ OR ‘Endoscopic Resection’ OR ‘Endoscopic Submucosal Dissection’ OR ‘EMR’ OR ‘ESD’)). Topics were assigned to subgroups of 2–3 panellists, based on their specific expertise. Each subgroup identified key controversies, evaluated the best evidence and proposed recommendations. Drafts were then discussed in virtual meetings and revised collectively. Although not a systematic review, the resulting consensus offers practical guidance for the endoscopic management of gastro‐duodenal‐rectal NETs, highlighting both current best practices and areas requiring further research.

## 3 RESULTS

### 3.1 Endoscopic techniques: practical choices for non‐experts

Selecting an appropriate endoscopic resection technique for gNETs, dNETs and rNETs is guided by factors such as tumour size and specific location, invasion depth and tumour grading.^6^, ^7^ Enteroendocrine cells, distributed from the stomach to the rectum, are essential for intestinal homeostasis, influencing nutrient sensing, absorption, barrier integrity and immune regulation.^8^ When neoplastic, their potential subepithelial growth – even in small tumours – can complicate endoscopic or local resection. This fact explains why standard endoscopic resection techniques using a simple snare‐based resection are often incomplete; as these tumours are often broad‐based or flattish, using a simple snare will not capture all of the hidden components of the tumour deep to its mucosal aspect. Snare polypectomy is thus often limited to less complex cases due to it frequently resulting in incomplete resection. Hence, there is a need for more advanced endoscopic resection techniques in most cases.

*Endoscopic mucosal resection* (EMR) starts with an injection to lift the lesion before resection, offering a more thorough removal suitable for larger lesions. Enhanced variants of EMR have been developed to increase the possibility of obtaining complete R0 endoscopic resection. ‘Underwater EMR’ (U‐EMR), which uses water to improve layer separation, provides better visualisation and separation of layers, but requires a specific setup and expertise. ‘Cap‐assisted’ EMR (c‐EMR) employs a cap for better suction and lesion security, facilitating more accurate snare placement, though it is limited to accessible lesion locations. ‘Circumferential Incision EMR’ (CI‐EMR) and ‘Circumferential Submucosal Incision EMR’ (CSI‐EMR), involving saline injection and targeted incisions, allow precise cutting suitable for superficially invasive tumours, but are more technically demanding and require higher skill. ‘Ligation‐assisted Modified EMR’ (L‐EMR) uses ligation to isolate the lesion, minimising damage to surrounding tissues, but it is complex and carries a risk of incomplete ligation.

*Endoscopic submucosal dissection* (ESD) is an advanced technique that starts with lesion demarcation and a submucosal cushion injection, followed by meticulous dissection under direct visualisation. ESD allows en‐bloc resection with precise margins, reducing recurrence, but is time‐consuming, carries a higher risk of perforation and haemorrhage (especially in the duodenum), and requires a high skill level.^9^

*Endoscopic full‐thickness resection* (EFTR) uses an over‐the‐scope clip and a polypectomy snare to secure and fully resect the lesion, sealing any resultant defect. EFTR is effective for lesions penetrating deeper layers, but has a high complication risk and should only be performed in specialised centres.

Each technique has specific advantages and is selected based on tumour characteristics and endoscopic expertise, aiming to balance efficacy and risk. Snare polypectomy is discouraged due to frequent incomplete resections. EMR, particularly in its modified forms, is generally preferred for non‐ampullary, non‐functional NETs. ESD is favoured for gastric and rectal lesions in experienced centres but should be used with caution for duodenal tumours due to higher complication risks. EFTR may be considered in selected cases involving deeper wall involvement. It is advisable to perform endoscopic tattooing to aid intraoperative localisation of the lesion in cases anticipated to undergo surgical resection. It is important to note that the endoscopic techniques discussed are applicable exclusively to non‐ampullary, non‐functional NETs; in functional or ampullary lesions, a surgical approach with oncologic intent remains the standard of care.

Patients with complex lesions (e.g., borderline in size or in whom deeper or indeterminate TNM‐stage lesions for endoscopic removal) may need a surgical approach, which underlines the need for NET multidisciplinary team decision‐making.

#### 3.1.1 Key points

- Snare polypectomy should be avoided for gNETs, dNETs and rNETs due to high incomplete resection rates.
- EMR and its advanced variants are preferred in most cases, while ESD should be considered for gastric and rectal lesions in expert centres.
- dNETs require a multidisciplinary approach, as ESD carries high perforation risks.
- EFTR is an alternative for deeper lesions in specialised settings.

### 3.2 What is the meaning of R1 resection

**FIGURE 1:**
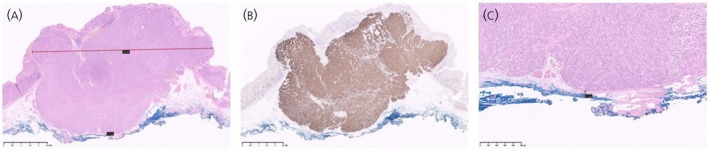
Pathologic findings. (A) (H&E) and (B) (Synaptophysin) show the endoscopic submucosal dissection (ESD) specimen for a gastric NET Type 3. The NET is confined to the submucosa and shows a pushing advancing border. Both lateral and deep margins are inked. The NET is well clear of both lateral margins. (C) (H&E) highlights the deep resection margin. The NET is <1 mm from the inked margin, but it is not at the margin, thus it is considered as clear (R0).

The assessment of the resection status following surgical/endoscopic removal of a NET is a key part of the histopathological examination. Traditionally, the term R1 resection refers to the presence of residual tumour cells at the resection margin on microscopic examination. This contrasts with an R0 resection, where no residual tumour cells are detected microscopically (Figure 1), and an R2 resection, where a macroscopic (visible) tumour remains after surgery/endoscopy.

While the overarching definition of R1 resection involves microscopic residual tumours at the resection margin, specific criteria can differ among cancer types, reflecting variations in tumour behaviour and anatomical considerations. In rectal adenocarcinoma, the circumferential resection margin (CRM) concept is pivotal. An R1 resection is defined when tumour cells are found within 1 millimetre of the CRM. This definition accounts for the propensity of rectal tumours to spread circumferentially and the importance of achieving clear margins to reduce local recurrence.^10^ The definition of R1 resection in pancreatic cancer is less standardised. Some guidelines, such as those from the British *Royal College of Pathologists*, define R1 as tumour cells within 1 millimetre of the margin, like rectal cancer.^11^ This approach considers the aggressive nature of pancreatic tumours and their tendency for early microscopic spread. For NETs, the definition of R1 resection typically aligns with the general standard of tumour cells being present microscopically at the resection margin.^12^

The clinical impact of R1 resection in gNETs, dNETs and rNETs remains under investigation, though recent studies suggest it may not significantly affect outcomes. In a recent series of 78 patients who underwent endoscopic resection for non‐ampullary dNETs, of the six patients who did not achieve curative resection, only one experienced disease recurrence during follow‐up.^13^ Similarly, the lack of complete R0 resection was not identified as a predictive factor for an increased risk of metastasis in a series of 105 rNETs, 25 of which had undergone incomplete resection.^14^ A retrospective analysis of patients with NETs in these regions found that tumour progression was rare following an R1 resection. The study concluded that microscopic positive margins after endoscopic resection did not adversely impact clinical outcomes, indicating that an R1 finding may not have substantial clinical significance.^15^ However, in the case of rNETs, given the small sample sizes of available studies and considering their potentially aggressive biological behaviour, prolonged follow‐up is advisable in patients with R1 resections to ensure timely detection of late recurrences. There is no absolute rule for deciding whether, when and how to proceed with reintervention following histological evidence of an R1 resection. Numerous factors must be considered, including the type of NET (Type 1 gNETs, for example, exhibit a more indolent behaviour that permits a conservative management approach in the case of an R1 resection), the Ki‐67 (even though standardised cut‐off values to guide decision‐making are lacking), the impact and risk of complications following reintervention, as well as the patient's age and comorbidities. The clinical significance of R1 resection in low‐grade NETs remains unclear, possibly reflecting their indolent nature; moreover, follow‐up is not standardised, thereby limiting our ability to determine the true impact of an R1 finding on clinical outcomes.

#### 3.2.1 Key points

- An R1 resection may not significantly impact prognosis in gNETs, dNETs and rNETs, but management should be individualised.
- Type 1 gNETs often allow conservative follow‐up, while dNETs require caution due to higher procedural risks.
- For rNETs, studies suggest incomplete resection is not a strong predictor of metastasis, supporting a more conservative approach in selected cases.
- Decision‐making should be guided by tumour type, proliferative index and patient factors, requiring a multidisciplinary discussion.

### 3.3 Pathological explanation and interpretation

Local endoscopic excisions of NETs often present unique challenges, so certain specimen‐specific features require additional scrutiny and detailed documentation in the pathology report. As for several other fields of pathology, a standardised pathology reporting approach is a key item to enable interdisciplinary decision‐making for treatment in a multidisciplinary approach, as it has been proposed for endoscopy.^16^ Key elements that integrate endoscopic and pathology findings typically include^12^:

1. Anatomical localisation (i.e., in the stomach: fundus, corpus, antrum, pre‐pyloric region, pylorus).
2. Tumour classification according to the WHO classification.^2^
3. Tumour grade (including exact numbers of mitotic rate and Ki‐67 proliferation index).
4. Tumour size (precise measurement of the tumour dimensions in mm).
5. Local invasion (mucosa/submucosa/muscularis propria).
6. Lymphovascular and perineural invasion.
7. Resection margins (distance to closest lateral and deep margin when feasible, R0 vs. R1).
8. Adjacent mucosa: absence of histological alterations or presence of H. Pylori‐related gastritis, the presence of CAG, the presence of enterochromaffin‐like‐cell hyperplasia and any other relevant finding.
9. Additional observations: invasion patterns (expansile vs. infiltrative), molecular subtyping if available (e.g., MEN1 mutations in type II gastric NETs, mTOR pathway activation).

While the morphology and grading of a NET form the backbone of the pathological diagnosis, additional data, ranging from the margin status, invasion patterns and background abnormalities, provide crucial supplementary information to tailor treatment strategies and follow‐up plans to the individual patient's risk profile.

Pathology report review at a referral centre is always recommended to ensure diagnostic accuracy and optimal patient management. Such a review becomes mandatory when missing or ambiguous data are present, as these can critically affect diagnosis, tumour grading, staging and therapeutic decisions.^17^ Notably, the rate of diagnostic discrepancies has been reported to be high, highlighting the importance of a standardised and centralised approach.^18^ In practice, revisions are generally warranted if any of the following conditions are met:

1. Omission of essential data: when key elements, such as tumour dimensions, margin status, depth of invasion, or tumour grade, are missing. These details are crucial for accurate staging and treatment planning.
2. Transcription or typographical errors: when errors in labelling, measurement units, or data entry occur, they can lead to diagnostic errors, especially if they affect the interpretation of resection status or tumour characteristics.
3. Insufficient clinical correlation: when there is a lack of correlation between the clinical information provided and the pathologic findings or when the clinical context is not clearly reflected in the report, a revision is advisable.

#### 3.3.1 Key points

- Standardised pathology reporting is essential for optimal gNETs, dNETs and rNETs management.
- Reports should include tumour localisation, classification, grade, size, invasion depth, lymphovascular involvement and resection margins.
- Pathology report revisions should be considered when key data are missing, errors are detected, or clinical correlation is inadequate.

### 3.4 Controversial issues in gastric NETs

#### 3.4.1 Prognostic role of Ki‐67 in type 1 gNETs

The Ki‐67 proliferation index is a well‐established marker used to assess the aggressiveness of NETs. In type 1 gNETs, which are typically small, the Ki‐67 index is generally low, reflecting their indolent nature.^3^, ^4^ However, variations in the Ki‐67 index can have prognostic implications. In a study discussing the assessment and prognostic role of the Ki‐67 labelling index in gastroenteropancreatic NENs, the authors highlighted that while type 1 gNETs usually present with a low Ki‐67 index, an elevated Ki‐67 may indicate a higher risk of aggressive behaviour and metastasis. In such cases, it is advisable to re‐evaluate the diagnostic classification carefully to confirm that the lesion is indeed a type 1 gNET and not misclassified as a type 3 tumour, which inherently carries a different biological behaviour and prognosis.^19^ Very rare G3 type 1 gNETs have been described, accounting for 1.6% of all type 1 gNETs in one series. Although G3 gNETs had a very poor prognosis, it was found that patient survival was longer than that related to NECs.^20^

In a multicentre study on type 1 gNETs, 137 patients were evaluated, with 75% classified as G1 and 25% as G2. Analysis indicated that male gender, tumour size and type, grading and the Ki‐67 index were associated with adverse outcomes. However, only tumour type and size remained significant in multivariate analysis, while the Ki‐67 index did not retain statistical significance in this analysis.^21^ Moreover, a retrospective multicentre series of 20 patients with metastatic type 1 gNETs reported a broad range of Ki‐67 values, from 1% to 20%. This observation implies that even tumours with a very low proliferative index can develop aggressive metastatic behaviour.^22^ In addition, a recent study involving 60 patients with type 1 gNETs with a median follow‐up of 5.8 years reported no association between the presence of lymph node metastases (evaluated by thoraco‐abdominal CT and functional imaging) and Ki‐67.^23^

Thus, the role of Ki‐67 in type 1 gNETs remains controversial, probably due to the limited number of tumours with high Ki‐67 levels in published studies. Although solid scientific evidence is lacking, caution is advisable when dealing with a G2 tumour and in the very rare G3 cases. Thus, an elevated Ki‐67 index may serve as an early indicator of potential aggressive behaviour, thereby influencing follow‐up intensity and therapeutic decisions.

##### Key points

- Unlike other gastrointestinal NETs, the Ki‐67 index does not have a clear prognostic role in type 1 gNETs.
- Ki‐67 may not always predict metastatic risk, but elevated values (grade 2) warrant closer follow‐up, given their potential for more aggressive behaviour.
- Grade 3 cases are exceedingly rare, yet they should be managed with the awareness of a potentially high risk of aggressiveness, despite the lack of specific data. These cases warrant comprehensive staging through radiological imaging and nuclear medicine examination to accurately assess disease extent and guide appropriate management.

#### 3.4.2 How to manage patients with multiple type 1 tumours

Type 1 gNETs are multiple in 63–72% of cases, making it technically challenging to achieve complete endoscopic resection of all visible lesions.^23^, ^24^ The optimal approach and the best resection strategy for multiple type 1 tumours are still not well established, owing to the absence of solid data supporting the most effective strategy to manage these patients.^25^ There are no randomised studies comparing an interventional approach (resecting all visible tumours) to a more conservative approach based on endoscopic surveillance.

As tumour size represents the main prognostic factor for the development of metastases and tumour size >10 mm has been identified as a cut‐off to recognise lesions with more aggressive behaviour, the minimal approach should be to suggest resection of tumours ≥10 mm according to ENETS guidelines.^4^, ^21^ However, the optimal therapeutic approach for sub‐centimetre lesions remains controversial. In a recent study by Chin et al., 29.5% of patients under surveillance progressed, requiring resection, suggesting that simple surveillance may not be safe in these patients.^26^ Interestingly, this study reported that high fasting serum gastrin concentrations were more likely related to disease progression. In contrast, a retrospective analysis showed that type 1 gNETs ≤10 mm were unlikely to develop clinically significant tumour progression. This suggests that the endoscopic surveillance interval could be safely extended to every 2–3 years in such patients.^27^ In addition, Esposito et al. found in a 10‐year follow‐up study that type 1 gNETs <5 mm could be followed up by non‐interventional endoscopic surveillance since none of these patients underwent disease progression after a median follow‐up of almost 4 years.^28^

Most type 1 gNETs are preceded or accompanied by linear or nodular ECL cell hyperplasia. Histologically, nodules >0.5 mm define NETs, distinguishing them from hyperplasia.^29^ Given the increased gNET risk in patients with CAG, regular endoscopic surveillance with biopsies is advised, though optimal intervals remain undefined.^30^ The timing of endoscopic surveillance depends on the extent of histological alterations and the presence of a family history of gastric cancer. In accordance with the recent MAPS III guidelines,^31^ patients with extensive endoscopic or histological changes are recommended to undergo high‐quality endoscopy every 3 years, whereas individuals with similar findings and a first‐degree relative with gastric cancer may benefit from a more intensive follow‐up at 1–2 year intervals (MAPS III).

Treatment with somatostatin analogues may be recommended in selected type 1 gNETs that cannot be safely managed by endoscopic surveillance or resection due to multiple or frequently recurring lesions, or when there is a large burden of polyps that continue to recur despite endoscopic follow‐up.^4^ The complete response rate ranges from 25% to 100%, but after therapy discontinuation, relapse is frequent.^32^ Netazepide, a gastrin receptor inhibitor, has been reported to result in a complete response rate in 30% of patients, albeit tumour relapse was observed after discontinuation of treatment.^33^ Further randomised clinical trials are required before implementing netazepide in clinical practice.

In cases of multiple type 1 gNETs, a personalised approach in the context of a multidisciplinary team is essential to guide the selection of endoscopic surveillance, endoscopic/surgical resection, or medical treatment in very selected cases.

##### Key points

- The optimal management of multiple type 1 gNETs remains uncertain. Resection is recommended for lesions ≥10 mm, while surveillance may be appropriate for smaller tumours, especially those <5 mm.
- High fasting gastrin levels may indicate progression risk. Somatostatin analogues or netazepide may be considered in selected cases, though recurrence is common after treatment discontinuation.

#### 3.4.3 The limit of endoscopic treatment in type 3 gNETs

The endoscopic management of type 3 gNETs remains unclear, with international guidelines providing conflicting recommendations. While ENETS supports endoscopic resection for tumours under 10 mm and low grade, provided metastases are ruled out and depth of invasion is assessed via endoscopic ultrasonography,^4^ other guidelines, such as those from ASGE,^34^ NANETS^35^ and NCCN,^36^ generally favour surgical treatment due to the high risk of lymph node metastases. The ESGE allows for endoscopic resection in select cases up to 20 mm with exclusive submucosal invasion.^7^ However, the endoscopic approach to type 3 gNETs has been increasingly adopted,^37^ driven by emerging data from recent studies. Although limited by small sample sizes and retrospective designs, these studies have reported that endoscopic management of small sporadic type 3 gNETs is feasible and safe. There are no clear criteria to determine when a type 3 gNET should be managed endoscopically or when surgical resection is necessary. To date, tumour size remains the only scientifically supported criterion, with a 1 cm threshold considered the cut‐off for endoscopic treatment. In the study by Exarchou et al.^38^ involving 45 patients with type 3 gNET, 36 of whom underwent endoscopic resection, a tumour size >1 cm was statistically associated with a significantly higher risk of poor patient outcomes. A similar finding was reported in the study by Hirasawa et al.^39^ where, in addition to tumour size, the depth of wall invasion was statistically associated with an increased risk of lymph node metastases. Outcomes were more favourable in cases where the tumour was confined to the mucosa or the submucosa with an invasion depth of <0.5 mm.

##### Key points

- Endoscopic resection may be considered for type 3 gNETs <10 mm (G1–G2, mucosa or superficial submucosa invasion <0.5 mm), preferably with Ki‐67 <10% as a precaution, despite limited supporting data.
- Surgical resection remains the preferred approach for tumours ≥10 mm or those invading beyond the superficial submucosa.
- Treatment decisions should be guided by tumour size, grade and depth of invasion, ideally within a multidisciplinary team.

### 3.5 Controversial issues in duodenal NETs

#### 3.5.1 Particular challenges facing a periampullary dNET

Pathological assessment of tissue samples from endoscopic resections is primarily aimed at confirming the diagnosis. However, the procedure's success can be influenced by factors such as tumour size, location (mucosal vs. submucosal) and the presence of surrounding structures. Sampling periampullary dNETs may present certain challenges due to the anatomical complexity. As stated above (see point 3.3), pathological reporting is to be structured to describe the key findings pivotal for clinical decision‐making. However, in the duodenal location, obtaining sufficient and representative tissue samples for histopathological evaluation can be difficult, potentially necessitating multiple sampling attempts. Limited tissue samples can hinder comprehensive histological evaluation, leading to potential underestimation of tumour grade or misclassification. In cases of very small almost complete excision at biopsy, it may make future identification difficult and render R1 resections infrequent. Moreover, tumour tissue investigation may need, other than Ki‐67, hormonal immunohistochemical characterisation since different functional types (such as gastrin‐producing or somatostatin‐producing or ordinary non‐functioning types) may have different risks of lymph node metastases.^40^ Finally, the ampullary location is a specific site for a particular type of NEN, the gangliocytic paraganglioma, which the WHO classification of NETs, 5th edition,^41^ has renamed composite gangliocytoma/neuroma and neuroendocrine tumour (CoGNET). This entity is characterised by a triphasic morphology (epithelioid NE, Schwannian‐like spindle and ganglion‐like) and may pose problems in the differential diagnosis with other periampullary neoplasms, including other NET types, paraganglioma, ganglioneuroma and gastrointestinal stromal tumours.

##### Key points

- Pathological assessment of endoscopic resections must ensure adequate tissue sampling, particularly for periampullary dNETs, to avoid underestimating tumour grade or misclassification. Multiple biopsies may be needed for representative sampling.
- Hormonal immunohistochemistry can aid in risk stratification based on functional type.
- In the ampullary region, differential diagnosis should consider CoGNET, a distinct entity requiring careful histopathological evaluation.

#### 3.5.2 Appropriate endoscopic assessment

Most dNETs occur in the first or second part of the duodenum, with those in the second part predominating in the ampullary region, the majority being discovered incidentally; more rarely, larger lesions could cause intestinal obstruction or jaundice (if periampullary or bleeding resulting in anaemia).^42^, ^43^ While gastrinomas are generally rare, their duodenal localisation is not infrequent, especially as part of MEN‐1, and these patients present with Zollinger‐Ellison syndrome.^43^ Somatostatin‐expressing NET and CoGNET are located almost exclusively in the ampullary region.^44^ Periampullary dNETs, including somatostatinomas, can occur in patients with neurofibromatosis type 1 (NF1).^43^, ^45^

The endoscopic morphological aspects of dNETs are similar to those in other gastrointestinal tract localisations, being usually single and small to medium‐sized^46^ sessile, erythematous, or pale lesions in the proximal duodenum or bulb or D1 to D2.^16^ In recent years, increasingly smaller lesions have been detected, owing to better mucosal visualisation with modern endoscopic tools. In a recent paper by Kim et al.^47^ magnifying endoscopy with narrow‐band imaging revealed that dNETs typically exhibit regular microsurface and microvascular patterns, along with the presence of thickened subepithelial vessels in approximately 68% of lesionlesions, features that may aid in differentiating these tumours from other subepithelial lesions; however, these findings are based on a small cohort of 22 lesions and should be interpreted with caution.

At endoscopy, lesions should be accurately measured and anatomically localised with a recording of their morphological aspects.^48^ EUS can also be used to assess TNM stage and exclude loco‐regional adenopathy; this is important for larger sporadic lesions (1 cm or greater) in planning therapy (endoscopic vs. surgery). Also, with a clinical suspicion of gastrinoma, EUS should always be performed regardless of size^43^, ^49^ due to the risk of loco‐regional metastasis, in addition, somatostatin‐receptor PET/CT is recommended. EUS is also important in assessing ampulla or periampullary NET and providing accurate localisation and TNM staging, and determining whether the pancreatic or biliary ducts are involved.

##### Key points

- dNETs are often incidental findings, with larger lesions potentially causing obstruction, jaundice, or bleeding.
- Periampullary dNETs may be associated with NF1 or MEN‐1; they are rarely amenable to endoscopic treatment due to their biological behaviour and anatomical location, usually requiring surgery. Accurate endoscopic measurement, localisation and morphological assessment are essential. EUS should be used for staging, evaluating loco‐regional adenopathy in lesions ≥1 cm, and assessing periampullary involvement.
- EUS, morphological imaging exams and somatostatin‐receptor PET/CT are recommended in suspected gastrinomas to evaluate metastatic risk.

#### 3.5.3 Limits of advanced endoscopic resections in the duodenum

The literature on the endoscopic resection of non‐ampullary duodenal tumours primarily focuses on non‐NENs, particularly adenomas, with limited data on dNETs. Most studies describe snare‐based techniques, mainly EMR, with success rates exceeding 90% for superficial lesions. Despite a high piecemeal resection rate (>30%), recurrence is uncommon and usually manageable with repeat endoscopic treatment.^9^

EMR is generally safe, with perforation rates below 5% and significant bleeding occurring in 10%–15% of cases.^9^ However, the duodenum's thin wall and rich vascularisation increase the risk of complications. Nonetheless, EMR‐related perforation remains relatively low at approximately 2%.^50^

ESD has been explored for superficial duodenal lesions, achieving higher en‐bloc resection rates (70%–80%) but with a perforation risk exceeding 30%, including delayed cases requiring surgery. Despite better complete resection rates than EMR, ESD has not shown superior long‐term outcomes or survival. Given these risks, the ESGE does not recommend routine ESD for superficial duodenal lesions.^9^

A recent meta‐analysis reported that duodenal ESD for non‐ampullary lesions achieves high en‐bloc and R0 resection rates (98% and 86%), but carries a significant perforation risk (8%), highlighting the importance of expert centres and careful patient selection.^51^

In a recent multicentre study^52^ involving 171 patients, EFTR for dNET demonstrated high efficacy (R0: 71.9%) and safety (severe adverse events: 1.8%), with very low recurrence rates (1.8%). Additionally, endoscopic vascular pattern assessment in dNETs showed high accuracy (93.3%) in predicting tumour grade and depth of invasion, highlighting its potential role in risk stratification. Given these findings, EFTR could represent a preferred endoscopic treatment for dNETs, particularly for lesions located distal to the proximal third of the duodenal bulb.

dNETs are highly heterogeneous, with their biological behaviour influenced by various factors.^53^ Given the risk of adverse events, dNET resection is best undertaken in high‐expertise centres. While multiple EMR and ESD variants have been reported in small cohorts, EFTR represents an emerging option, though current evidence is still limited.^54^ Surgery should be considered when endoscopic resection carries a high risk of complications or R1 margins. All duodenal resections require careful multidisciplinary planning to balance resection success with procedural risks and surgical alternatives.^55^

##### Key points

- Endoscopic resection of non‐ampullary dNETs is challenging due to the thin duodenal wall and rich vascularisation.
- EMR is the preferred technique, with high success rates and acceptable complication risks, though piecemeal resection is common.
- ESD achieves higher en‐bloc resection rates but carries an unacceptably high perforation risk and is not routinely recommended.
- Resections should be performed in experienced centres, and high‐risk cases should be evaluated for surgery. EFTR is an emerging option, but evidence remains limited.

#### 3.5.4 How to manage small incidental dNETs

dNETs pose particular challenges in management due to the inherent heterogeneity and the lack of prospective outcome data, especially in small incidentally discovered lesions.^56^

For incidentally found non‐functional lesions, therapy will depend on: the size and grade of the tumour^48^; ease of access for endoscopic resection (post‐pyloric or periampullary tumours may pose certain challenges); TNM stage (depth of invasion in relation to the deep muscle layer, assessed at EUS); possibility of lymphadenopathy and/or metastatic spread; tumour grade. With advancing techniques, endoscopic resection is increasingly effective for selected dNETs >10 mm in patients with non‐metastatic disease,^57^ though treatment decisions should always be made in a multidisciplinary setting.

For very small lesions <10 mm, patient age, and performance status need to be considered. It is difficult to ascribe definite rules to dNET management,^58^ but in general, for tumours <10 mm that do not involve the ampulla and in whom loco‐regional spread is not present or likely, patients could have surveillance or endoscopic resection (as described earlier).^4^ EMR or ESD is often preferred to cap‐assisted techniques or full‐thickness endoscopic resections that are sometimes difficult in these localisations. The choice of technique will depend on local expertise. A recent retrospective study including 56 patients with non‐ampullary duodenal lesions (28 of whom had NETs) demonstrated similar efficacy between EMR and ESD in achieving radical resection with R0 margins. However, the study reported a higher complication rate for ESD (duodenal perforation occurred in 10% of patients), which is therefore recommended only for larger lesions, following prior assessment of lesion infiltration using EUS.^59^ Surgery is rarely indicated for small (<10 mm), non‐functional dNETs; however, SEER data analysis (2010–2015) reported its use in up to 46% of cases, even for lesions <10 mm.^60^ This interesting analysis included 465 patients treated surgically or endoscopically for a mixture of dNETs and showed that when controlling for confounding factors, endoscopic resection of dNETs had similar survival to surgical resection, irrespective of tumour size and also lymph node status.^60^ In another study, 22 of 69 patients with ≤10 mm, grade 1, non‐functioning, non‐ampullary dNETs underwent a ‘watch and wait’ or surveillance, with only one showing a mild local increase in size.^46^ While some have challenged this conservative approach,^61^ surveillance may be appropriate in a subset of patients with dNETs.

##### Key points

- Surveillance may be considered for non‐functional, non‐ampullary dNETs ≤10 mm (G1, no EUS evidence of invasion or lymphadenopathy), particularly when endoscopic resection is not feasible. Follow‐up intervals are not well defined, but annual endoscopy and imaging appear reasonable.
- EMR is preferred for resection, while ESD is reserved for larger lesions due to higher perforation risk.
- Surgery is rarely needed for small dNETs; the type of intervention should be individualised based on tumour characteristics.

### 3.6 Controversial issues in rectal NETs

#### 3.6.1 Why and how to recognise rNET before endoscopic removal

rNETs are predominantly sessile, submucosal tumours with a smooth, regular surface and a characteristic yellowish coloration, often described as lipoma‐like. In rare cases, they may appear semi‐pedunculated or as multiple lesions, complicating differentiation from hyperplastic or adenomatous polyps. Larger lesions (>1 cm) may exhibit irregular surfaces, hyperaemia, ulceration, or central depression, suggesting more aggressive behaviour.^5^, ^62^ Given the high risk of R1 resection, biopsies or snare polypectomy should be avoided for suspected NETs.^55^ Instead, direct resection using an appropriate endoscopic technique, such as EMR or ESD, is essential to minimise this risk.

A recent comparative analysis^63^ of m‐EMR and ESD for rNETs reported an R0 resection rate of 90.0% for m‐EMR and 82.3% for ESD, with en‐bloc resection achieved in 100% of cases. No significant difference was observed in post‐procedural bleeding (5.5% for m‐EMR vs. 2.8% for ESD). Additionally, the risk of R1 resection increased with tumour size, with lesions ≥5 mm significantly associated with incomplete histologic resection (OR: 1.25, *p* = .008), highlighting the challenge of achieving negative margins in larger tumours.

A study including 100 patients found that in cases of non‐R0 resection, residual tumour tissue was present in 43% of patients after salvage endoscopic resection. Consequently, systematic scar resection (via endoscopic full‐thickness resection or ESD) appears necessary, as these procedures have demonstrated an R0 resection rate close to 100%.^64^

Another recent study^65^ identified tumour size, resection margin involvement and angiolymphatic invasion as key risk factors for lymph node metastasis, with increasing rates as risk factors accumulate. Despite this, long‐term outcomes were favourable, with no significant difference in 5‐year recurrence‐free survival (98.7% vs. 99%) or overall survival (100% vs. 99.5%) between high‐ and low‐risk groups.

A study on small rNETs^66^ evaluated endoscopic strategies for achieving R0 resection in tumours ≤10 mm. The R0 resection rate for accidental diagnostic biopsy by cold forceps was 100% for rNETs 2–3 mm but dropped significantly for larger lesions (34.3% for 4–5 mm and 0% for 6–10 mm). In contrast, EMR and ESD showed comparable efficacy, with R0 rates of 87.5% and 93.8% for rNETs 6–10 mm, respectively. These findings suggest that while biopsy may be sufficient for very small tumours (≤3 mm), it should be avoided for larger ones due to the high risk of incomplete resection and residual disease.

Focusing on the management of R1 resections and the consideration of salvage therapy, a multicentre study^67^ assessed the clinical significance of positive resection margins in rNETs ≤20 mm after endoscopic resection. In a cohort of 527 patients, 34.3% had positive margins, correlating with worse disease‐free survival. Independent predictors included tumour size >1.5 cm, low rectal location, neutrophil‐to‐lymphocyte ratio >4.4 and resection technique. EMR and its variants were linked to higher rates of incomplete resection, while ESD showed the lowest R1 rate, supporting its role in achieving R0 resection. Salvage treatment significantly improved outcomes, with repeat EMR or ESD showing efficacy comparable to radical surgery, supporting less invasive strategies in selected cases.

In addition, the potential prognostic role of endoscopic vascular patterns in rNETs has been recently proposed, showing promising accuracy in predicting tumour grade and invasiveness.^68^

##### Key points

- Endoscopic resection is preferred for rNETs <1 cm and indicated in select cases for tumours 1–2 cm based on risk factors and local expertise.
- Switching to ESD or EFTR may be preferable for tumours >1.5 cm to reduce the risk of positive margins.
- Snare polypectomy and biopsies should be avoided for lesions >3 mm due to the risk of incomplete resection.
- Salvage endoscopic resection is effective for R1 cases.
- Vascular pattern assessment (V1/V2) aids risk stratification, correlating with tumour grade and invasion depth.
- Multidisciplinary management is essential for treatment optimisation.

## 4 CONCLUSIONS

The endoscopic management of gNETs, dNETs and rNETs is marked by key areas of uncertainty and variability in clinical practice. This ENETS position paper highlights the importance of a tailored and multidisciplinary strategy that accounts for tumour size, location and biological behaviour (Table 2). EMR and its advanced variants remain the preferred approach for most small lesions, while ESD and EFTR may be appropriate in selected cases, particularly when deeper invasion is suspected. Notably, an R1 resection does not necessarily imply a poor prognosis and, in select scenarios, may be managed conservatively. Accurate and standardised pathological assessment – including grading, invasion depth and background mucosa – is essential for clinical decision‐making. Controversies persist regarding the prognostic role of Ki‐67 in type 1 gNETs, the management of multiple gastric lesions, and the role of endoscopic resection in type 3 gNETs and dNETs. For rNETs, recognising subtle endoscopic features and selecting optimal resection techniques are crucial to achieving complete removal and minimising recurrence. Standardised surveillance protocols are urgently needed for type 1 gNETs, particularly given the limited prospective data. The recent ESGE MAPS III guidelines^31^ highlight the role of high‐quality endoscopy with chromo‐endoscopy in advanced CAG, potentially reducing the need for biopsy mapping. This paradigm shift may also impact follow‐up strategies in patients with a history of gNET. Additional research is essential to refine treatment algorithms and support evidence‐based, patient‐centred management.

**TABLE 2:** Highlights of controversial issues in managing gastric, duodenal, and rectal NETs.

Multidisciplinary evaluation is essential
The management of gastric, duodenal, and rectal NETs must be individualised and discussed in expert multidisciplinary teams, considering tumour type, size, location, grade, and procedural risks.
Endoscopic resection is often feasible and effective
Advanced endoscopic techniques (e.g., modified EMR, ESD, EFTR) allow curative resection in most gNETs, dNETs, and rNETs. Snare polypectomy should be avoided due to high rates of incomplete resection.
R1 resection may not always warrant further intervention
Evidence suggests that microscopic positive margins (R1) do not consistently predict recurrence or metastasis, particularly in type 1 gNETs and small rNETs, supporting conservative management in selected cases.
The role of Ki‐67 in type 1 gNETs is controversial
Although elevated Ki‐67 may suggest more aggressive behaviour, its prognostic significance in type 1 gNETs is limited, and tumour size appears to be stronger predictors of outcome.
Multiple type 1 gNETs pose unique challenges
Management should be personalised; resection is generally advised for lesions ≥10 mm, while smaller lesions may be monitored. Somatostatin analogs are an option in highly selected cases.
Endoscopic treatment of type 3 gNETs requires caution
Endoscopic resection may be considered for small (<10 mm), low‐grade, superficial tumours, but surgery remains the standard for larger or deeply invasive lesions.
Duodenal NETs are technically challenging
Due to thin walls and vascularity, duodenal resections (especially ESD) carry high risks. EMR is preferred for small non‐ampullary lesions; EFTR is promising for selected cases in expert centres.
Small, incidental dNETs may not require immediate intervention
For non‐functional, ≤10 mm, non‐ampullary dNETs without high‐risk features, surveillance is a reasonable option. However, surgical overtreatment is still reported in clinical practice.
rNETs require precise recognition and technique selection
Biopsy or polypectomy should be avoided for lesions >3 mm. EMR or ESD should be tailored based on size and invasion risk. Salvage endoscopic resection is effective in R1 cases.
Key areas for future research remain unmet
High‐priority unmet needs include the definition of standardised follow‐up protocols (especially for type 1 gNETs), the prognostic value of Ki‐67 and other biomarkers, the role of vascular pattern analysis, the optimal management of multiple gastric lesions, and prospective data on the safety and efficacy of endoscopic resection for type 3 gNETs and dNETs.

## AUTHOR CONTRIBUTIONS

**Francesco Panzuto:** Conceptualization; writing – original draft; methodology; validation; writing – review and editing; project administration; supervision. **Dermot O'Toole:** Writing – original draft; conceptualization; writing – review and editing. **Günter Klöppel:** Writing – original draft; writing – review and editing. **Ulrich Peter Knigge:** Writing – original draft; writing – review and editing. **Günter Josef Krejs:** Writing – original draft; writing – review and editing. **Marina Tsoli:** Writing – original draft; writing – review and editing. **Marco Volante:** Writing – original draft; writing – review and editing. **Tu Vinh Luong:** Conceptualization; methodology; writing – original draft; writing – review and editing; supervision.

## CONFLICT OF INTEREST STATEMENT

Tu Vinh Luong has received honoraria from AAA (UK & Ireland) Limited. All other authors declare no conflicts of interest.

## Data Availability

Data sharing not applicable to this article as no datasets were generated or analysed during the current study.

## References

[1] Abboud Y , Shah A , Sutariya R , et al. Gastroenteropancreatic neuroendocrine tumor incidence by sex and age in the US. JAMA Oncol. 2025;11(3):345‐349. doi:10.1001/jamaoncol.2024.5937 39821008 PMC11926624

[2] Rindi G , Mete O , Uccella S , et al. Overview of the 2022 WHO classification of neuroendocrine neoplasms. Endocr Pathol. 2022;33(1):115‐154. doi:10.1007/s12022-022-09708-2 35294740

[3] Lamberti G , Panzuto F , Pavel M , et al. Gastric neuroendocrine neoplasms. Nat Rev Dis Primers. 2024;10(1):25. doi:10.1038/s41572-024-00508-y 38605021

[4] Panzuto F , Ramage J , Pritchard DM , et al. European neuroendocrine tumor society (ENETS) 2023 guidance paper for gastroduodenal neuroendocrine tumours (NETs) G1‐G3. J Neuroendocrinol. 2023;35(8):e13306. doi:10.1111/jne.13306 37401795

[5] Rinke A , Ambrosini V , Dromain C , et al. European neuroendocrine tumor society (ENETS) 2023 guidance paper for colorectal neuroendocrine tumours. J Neuroendocrinol. 2023;35(6):e13309. doi:10.1111/jne.13309 37345509

[6] Esposito G , Dell'Unto E , Ligato I , Marasco M , Panzuto F . The meaning of R1 resection after endoscopic removal of gastric, duodenal and rectal neuroendocrine tumors. Expert Rev Gastroenterol Hepatol. 2023;17(8):785‐793. doi:10.1080/17474124.2023.2242261 37497604

[7] Deprez PH , Moons LMG , O'Toole D , et al. Nieveen van Dijkum E, Blay JY, van Hooft JE. Endoscopic management of subepithelial lesions including neuroendocrine neoplasms: European Society of Gastrointestinal Endoscopy (ESGE) guideline. Endoscopy. 2022;54(4):412‐429. doi:10.1055/a-1751-5742 35180797

[8] Nwako JG , McCauley HA . Enteroendocrine cells regulate intestinal homeostasis and epithelial function. Mol Cell Endocrinol. 2024;593:112339. doi:10.1016/j.mce.2024.112339 39111616 PMC11401774

[9] Pimentel‐Nunes P , Dinis‐Ribeiro M , Ponchon T , et al. Endoscopic submucosal dissection: European Society of Gastrointestinal Endoscopy (ESGE) guideline. Endoscopy. 2015;47(9):829‐854. doi:10.1055/s-0034-1392882 26317585

[10] Wittekind C , Compton C , Quirke P , et al. A uniform residual tumor (R) classification: integration of the R classification and the circumferential margin status. Cancer. 2009;115(15):3483‐3488. doi:10.1002/cncr.24320 19536900

[11] Campbell F , Cairns A , Duthie F , Feakins R . Dataset for Histopathological Reporting of Carcinomas of the Pancreas, Ampulla of Vater and Common Bile Duct. The Royal College of Pathologists; 2019.

[12] Luong TV , Watkins J , Chakrabarty B , Wang LM . Dataset for Histopathological Reporting of Neuroendocrine Neoplasms of the Gastroenteropancreatic Tract. The Royal College of Pathologists; 2019.

[13] Wang Y , Ren Z , Shen YH , et al. Long‐term outcomes of endoscopic resection for well‐differentiated nonampullary duodenal neuroendocrine tumors. Gastrointest Endosc. 2024;100(3):481‐491.e6. doi:10.1016/j.gie.2024.02.017 38431107

[14] Busan Ulsan Gyeongnam Intestinal Study Group Society (BIGS) , Goo JJ , Baek DH , et al. Clinical outcomes and risk factors associated with poor prognosis after endoscopic resection of 10–20 mm rectal neuroendocrine tumors: a multicenter, retrospective study of 10‐year experience. Surg Endosc. 2023;37(7):5196‐5204. doi:10.1007/s00464-023-09999-4 36947224

[15] Dell'Unto E , Marasco M , Mosca M , et al. Clinical outcome of patients with gastric, duodenal, or rectal neuroendocrine tumors after incomplete endoscopic resection. J Clin Med. 2024;13(9):2535. doi:10.3390/jcm13092535 38731064 PMC11084244

[16] Borbath I , Pape UF , Deprez PH , et al. Members of the advisory Board of the European Neuroendocrine Tumor Society (ENETS). ENETS standardised (synoptic) reporting for endoscopy in neuroendocrine tumors. J Neuroendocrinol. 2022;34(3):e13105. doi:10.1111/jne.13105 35233848

[17] Marasco M , Magi L , Rogges E , et al. Utility of histopathological revision in the management of gastro‐entero‐pancreatic neuroendocrine neoplasia. Endocrine. 2023;82(2):435‐441. doi:10.1007/s12020-023-03418-3 37338723 PMC10543798

[18] Waked B , De Maeyer F , Carton S , et al. Quality of pathology reporting and adherence to guidelines in rectal neuroendocrine neoplasms: a Belgian national study. Acta Clin Belg. 2022;77(5):823‐831. doi:10.1080/17843286.2021.1985806 34607538

[19] Klöppel G , La Rosa S . Ki67 labeling index: assessment and prognostic role in gastroenteropancreatic neuroendocrine neoplasms. Virchows Arch. 2018;472(3):341‐349. doi:10.1007/s00428-017-2258-0 29134440

[20] Vanoli A , La Rosa S , Miceli E , et al. Prognostic evaluations tailored to specific gastric neuroendocrine neoplasms: analysis of 200 cases with extended follow‐up. Neuroendocrinology. 2018;107(2):114‐126. doi:10.1159/000489902 29895024

[21] Panzuto F , Campana D , Massironi S , et al. Tumour type and size are prognostic factors in gastric neuroendocrine neoplasia: a multicentre retrospective study. Dig Liver Dis. 2019;51(10):1456‐1460. doi:10.1016/j.dld.2019.04.016 31175013

[22] Grozinsky‐Glasberg S , Thomas D , Strosberg JR , et al. Metastatic type 1 gastric carcinoid: a real threat or just a myth? World J Gastroenterol. 2013;19(46):8687‐8695. doi:10.3748/wjg.v19.i46.8687 24379587 PMC3870515

[23] Ravizza D , Giunta M , Sala I , et al. Gastric neuroendocrine tumors: 20‐year experience in a reference center. J Neuroendocrinol. 2024;36(12):e13440. doi:10.1111/jne.13440 39191460

[24] Chen YY , Guo WJ , Shi YF , et al. Management of type 1 gastric neuroendocrine tumors: an 11‐year retrospective single‐center study. BMC Gastroenterol. 2023;23(1):440. doi:10.1186/s12876-023-03079-6 38097952 PMC10722838

[25] Panzuto F , Magi L , Esposito G , Rinzivillo M , Annibale B . Comparison of endoscopic techniques in the Management of Type I Gastric Neuroendocrine Neoplasia: a systematic review. Gastroenterol Res Pract. 2021;2021:6679397. doi:10.1155/2021/6679397 33859684 PMC8026302

[26] Chin JL , O'Connell J , Muldoon C , et al. Selective resection of type 1 gastric neuroendocrine neoplasms and the risk of progression in an endoscopic surveillance Programme. Dig Surg. 2021;38(1):38‐45. doi:10.1159/000510962 33260173

[27] Exarchou K , Hu H , Stephens NA , et al. Endoscopic surveillance alone is feasible and safe in type I gastric neuroendocrine neoplasms less than 10 mm in diameter. Endocrine. 2022;78(1):186‐196. doi:10.1007/s12020-022-03143-3 35895180 PMC9474380

[28] Esposito G , Cazzato M , Rinzivillo M , et al. Management of type‐I gastric neuroendocrine neoplasms: a 10‐years prospective single centre study. Dig Liver Dis. 2022;54(7):890‐895. doi:10.1016/j.dld.2021.11.012 34903498

[29] La Rosa S , Uccella S . Endocrine pathology. Encyclopedia of Pathology; Springer Cham. 2022. doi:10.1007/978-3-030-62345-6

[30] Annibale B , Azzoni C , Corleto VD , et al. Atrophic body gastritis patients with enterochromaffin‐like cell dysplasia are at increased risk for the development of type I gastric carcinoid. Eur J Gastroenterol Hepatol. 2001;13(12):1449‐1456. doi:10.1097/00042737-200112000-00008 11742193

[31] Dinis‐Ribeiro M , Libânio D , Uchima H , et al. Management of epithelial precancerous conditions and early neoplasia of the stomach (MAPS III): European Society of Gastrointestinal Endoscopy (ESGE), European helicobacter and microbiota study group (EHMSG) and European Society of Pathology (ESP) guideline update 2025. Endoscopy. 2025;57:504‐554. doi:10.1055/a-2529-5025 40112834

[32] Rossi RE , Invernizzi P , Mazzaferro V , Massironi S . Response and relapse rates after treatment with long‐acting somatostatin analogs in multifocal or recurrent type‐1 gastric carcinoids: a systematic review and meta‐analysis. United Eur Gastroenterol J. 2020;8(2):140‐147. doi:10.1177/2050640619890465 PMC7079271 32213066

[33] Boyce M , Moore AR , Sagatun L , et al. Netazepide, a gastrin/cholecystokinin‐2 receptor antagonist, can eradicate gastric neuroendocrine tumours in patients with autoimmune chronic atrophic gastritis. Br J Clin Pharmacol. 2017;83(3):466‐475. doi:10.1111/bcp.13146 27704617 PMC5306499

[34] Standards of Practice Committee , Faulx AL , Kothari S , et al. The role of endoscopy in subepithelial lesions of the GI tract. Gastrointest Endosc. 2017;85(6):1117‐1132. doi:10.1016/j.gie.2017.02.022 28385194

[35] Kulke MH , Anthony LB , Bushnell DL , et al. North American neuroendocrine tumor society (NANETS). NANETS treatment guidelines: well‐differentiated neuroendocrine tumors of the stomach and pancreas. Pancreas. 2010;39(6):735‐752. doi:10.1097/MPA.0b013e3181ebb168 20664472 PMC3100728

[36] www.nccn.org/professionals/physician_gls/pdf/neuroendocrine.pdf, accessed 22 March 2025.

[37] Dell'Unto E , Esposito G , Rinzivillo M , Marasco M , Annibale B , Panzuto F . Type 3 gastric neuroendocrine neoplasms: the rising promise of conservative endoscopic management. Front Med (Lausanne). 2024;11:1327864. doi:10.3389/fmed.2024.1327864 38357651 PMC10864619

[38] Exarchou K , Kamieniarz L , Tsoli M , et al. Is local excision sufficient in selected grade 1 or 2 type III gastric neuroendocrine neoplasms? Endocrine. 2021;74(2):421‐429. doi:10.1007/s12020-021-02775-1 34120313

[39] Hirasawa T , Yamamoto N , Sano T . Is endoscopic resection appropriate for type 3 gastric neuroendocrine tumors? Retrospective multicenter study. Dig Endosc. 2021;33(3):408‐417. doi:10.1111/den.13778 32578248

[40] Vanoli A , Grami O , Klersy C , et al. Ampullary neuroendocrine neoplasms: identification of prognostic factors in a multicentric series of 119 cases. Endocr Pathol. 2022;33(2):274‐288. doi:10.1007/s12022-022-09720-6 35553369 PMC9135850

[41] WHO Classification of Tumours Editorial Board . Endocrine and neuroendocrine tumours. n.d. Available from: https://publications.iarc.who.int/645

[42] Delle Fave G , Kwekkeboom DJ , Van Cutsem E , et al. ENETS consensus guidelines for the management of patients with gastroduodenal neoplasms. Neuroendocrinology. 2012;95(2):74‐87. doi:10.1159/000335595 22262004

[43] Hoffmann KM , Furukawa M , Jensen RT . Duodenal neuroendocrine tumors: classification, functional syndromes, diagnosis and medical treatment. Best Pract Res Clin Gastroenterol. 2005;19(5):675‐697. doi:10.1016/j.bpg.2005.05.009 16253893

[44] Luna IE , Monrad N , Binderup T , et al. Somatostatin‐immunoreactive pancreaticoduodenal neuroendocrine neoplasms: twenty‐three cases evaluated according to the WHO 2010 classification. Neuroendocrinology. 2016;103(5):567‐577. doi:10.1159/000441605 26505735

[45] Stamm B , Hedinger CE , Saremaslani P . Duodenal and ampullary carcinoid tumors. A report of 12 cases with pathological characteristics, polypeptide content and relation to the MEN I syndrome and von Recklinghausen's disease (neurofibromatosis). Virchows Arch A Pathol Anat Histopathol. 1986;408(5):475‐489. doi:10.1007/BF00705301 2869609

[46] Exarchou K , Moore AR , Smart HL , Duckworth CA , Howes N , Pritchard DM . A “watch and wait” strategy involving regular endoscopic surveillance is safe for many patients with small, sporadic, grade 1, non‐ampullary, non‐functioning duodenal neuroendocrine Tumours. Neuroendocrinology. 2021;111(8):764‐774. doi:10.1159/000511613 32937631

[47] Kim GH , Yi K , Joo DC , Lee MW , Jeon HK , Lee BE . Magnifying endoscopy with narrow‐band imaging for duodenal neuroendocrine tumors. J Clin Med. 2023;12(9):3106. doi:10.3390/jcm12093106 37176547 PMC10179496

[48] The Paris endoscopic classification of superficial neoplastic lesions: esophagus, stomach, and colon: November 30 to December 1, 2002. Gastrointest Endosc. 2003;58(6 Suppl):S3‐S43. doi:10.1016/s0016-5107(03)02159-x 14652541

[49] Zogakis TG , Gibril F , Libutti SK , et al. Management and outcome of patients with sporadic gastrinoma arising in the duodenum. Ann Surg. 2003;238(1):42‐48. doi:10.1097/01.SLA.0000074963.87688.31 12832964 PMC1422657

[50] ASGE Technology Committee , Hwang JH , Konda V , et al. Endoscopic mucosal resection. Gastrointest Endosc. 2015;82(2):215‐226. doi:10.1016/j.gie.2015.05.001 26077453

[51] Rimondi A , Dell'Unto E , Morais R , et al. Outcomes and safety of duodenal endoscopic submucosal dissection for non‐ampullary lesion: a systematic review and meta‐analysis. Gastrointest Endosc. 2025;S0016‐5107(25)01509–3. doi:10.1016/j.gie.2025.03.1332 40210009

[52] Wannhoff A , Nabi Z , Moons LMG , et al. International, multicenter analysis of endoscopic full‐thickness resection of duodenal neuroendocrine tumors. Am J Gastroenterol. 2025. doi:10.14309/ajg.0000000000003409 40079474

[53] Massironi S , Campana D , Partelli S , et al. Heterogeneity of duodenal neuroendocrine tumors: an Italian multi‐center experience. Ann Surg Oncol. 2018;25(11):3200‐3206. doi:10.1245/s10434-018-6673-5 30054824

[54] Chacchi‐Cahuin R , Despott EJ , Lazaridis N , et al. Endoscopic Management of Gastro‐Entero‐Pancreatic Neuroendocrine Tumours: an overview of proposed resection and ablation techniques. Cancers (Basel). 2024;16(2):352. doi:10.3390/cancers16020352 38254841 PMC10814323

[55] Panzuto F , Parodi MC , Esposito G , et al. Endoscopic management of gastric, duodenal and rectal NETs: position paper from the Italian Association for Neuroendocrine Tumors (Itanet), Italian Society of Gastroenterology (SIGE), Italian Society of Digestive Endoscopy (SIED). Dig Liver Dis. 2024;56(4):589‐600. doi:10.1016/j.dld.2023.12.015 38216439

[56] Scherübl H , Jensen RT , Cadiot G , Stölzel U , Klöppel G . Neuroendocrine tumors of the small bowels are on the rise: early aspects and management. World J Gastrointest Endosc. 2010;2(10):325‐334. doi:10.4253/wjge.v2.i10.325 21160582 PMC2998818

[57] Rossi RE , Masoni B , Massironi S , et al. Endoscopic resection for duodenal neuroendocrine neoplasms between 10 and 20 mm‐a systematic review and meta‐analysis. J Clin Med. 2024;13(5):1466. doi:10.3390/jcm13051466 38592317 PMC10934162

[58] Margonis GA , Samaha M , Kim Y , et al. A multi‐institutional analysis of duodenal neuroendocrine tumors: tumor biology rather than extent of resection dictates prognosis. J Gastrointest Surg. 2016;20(6):1098‐1105. doi:10.1007/s11605-016-3135-x 27008594

[59] Jiang Y , Yang Z , Lin C , Yang J , Zheng X . Endoscopic resection for non‐ampullary duodenal subepithelial lesions: a retrospective cohort study. Int J Colorectal Dis. 2024;39(1):122. doi:10.1007/s00384-024-04698-5 39085622 PMC11291567

[60] Mirzaie S , Park JY , Mederos MA , Girgis MD . Surgical and endoscopic resection of duodenal neuroendocrine tumors have similar disease‐specific survival outcome. J Gastrointest Surg. 2023;27(11):2365‐2372. doi:10.1007/s11605-023-05800-y 37552388 PMC10661787

[61] Ratnayake GM , Srirajaskanthan R , Luong T , Gnanasegaran G , Toumpanakis C . Duodenal neuroendocrine neoplasms with unexpectedly aggressive behaviour: challenging the “watch and wait” approach. J Neuroendocrinol. 2022;34(1):e13067. doi:10.1111/jne.13067 34914146

[62] Reumkens A , Sastrowijoto P , Grabsch HI , et al. Epidemiological, clinical and endoscopic characteristics of colorectal neuroendocrine neoplasms: a population‐based study in The Netherlands. Endosc Int Open. 2022;10(7):E940‐E951. doi:10.1055/a-1793-9057 35845029 PMC9286769

[63] Kitagawa Y , Suzuki T , Miyakawa A , et al. Comparison of endoscopic submucosal dissection and modified endoscopic mucosal resection for rectal neuroendocrine tumors. Sci Rep. 2025;15(1):5424. doi:10.1038/s41598-024-82082-7 39948094 PMC11825951

[64] Cheminel L , Lupu A , Wallenhorst T , et al. Systematic resection of the visible scar after incomplete endoscopic resection of rectal neuroendocrine tumors. Am J Gastroenterol. 2024;119(2):378‐381. doi:10.14309/ajg.0000000000002516 37734341

[65] Park SS , Kim BC , Lee DE , et al. Stratification of risk for lymph node metastasis and long‐term oncologic outcomes in patients initially treated by endoscopic resection for rectal neuroendocrine tumors. Gastrointest Endosc. 2025;101(6):S0016‐5107(24)03746–5. doi:10.1016/j.gie.2024.11.036 39608591

[66] Wang J , Zhang X , Chen K , et al. Optimization of endoscopic treatment strategies for R0 resection of rectal neuroendocrine tumors smaller than 10 mm. Clin Res Hepatol Gastroenterol. 2024;48(9):102469. doi:10.1016/j.clinre.2024.102469 39332765

[67] Duan M , Liu Z , Qiao Y , et al. Clinical significance of positive resection margin for patients with rectal neuroendocrine tumors within 20 mm following initial endoscopic resection: a multi‐center study. Eur J Surg Oncol. 2024;50(11):108651. doi:10.1016/j.ejso.2024.108651 39243695

[68] Zheng Y , Hu Y , Li Y , Cui C , Wang X , Ji R . A new endoscopic tumor grading for rectal neuroendocrine tumors: correlation of vascular pattern with histopathology. Dig Liver Dis. 2025;57(3):782‐787. doi:10.1016/j.dld.2024.12.002 39690021

